# Intravascular NK/T-Cell Lymphoma: What We Know about This Diagnostically Challenging, Aggressive Disease

**DOI:** 10.3390/cancers14215458

**Published:** 2022-11-06

**Authors:** Magda Zanelli, Paola Parente, Francesca Sanguedolce, Maurizio Zizzo, Andrea Palicelli, Alessandra Bisagni, Illuminato Carosi, Domenico Trombetta, Luca Mastracci, Linda Ricci, Saverio Pancetti, Giovanni Martino, Giuseppe Broggi, Rosario Caltabiano, Alberto Cavazza, Stefano Ascani

**Affiliations:** 1 Pathology Unit, Azienda USL-IRCCS di Reggio Emilia, 42123 Reggio Emilia, Italy; 2 Pathology Unit, Fondazione IRCCS Casa Sollievo della Sofferenza, 71013 San Giovanni Rotondo, Italy; 3 Pathology Unit, Policlinico Riuniti, University of Foggia, 71122 Foggia, Italy; 4 Surgical Oncology Unit, Azienda USL-IRCCS di Reggio Emilia, 42123 Reggio Emilia, Italy; 5 Laboratory Oncology, Fondazione IRCCS Casa Sollievo della Sofferenza San Giovanni Rotondo, 71013 San Giovanni Rotondo, Italy; 6 Anatomic Pathology, Ospedale Policlinico San Martino IRCCS Genova, 16132 Genova, Italy; 7 Pathology Unit, Azienda Ospedaliera Santa Maria di Terni, University of Perugia, 05100 Terni, Italy; 8 Pathology Unit, Humanitas University, Pieve Emanuele, 20072 Milan, Italy; 9 Pathology Unit, Humanitas Research Hospital-IRCCS, Rozzano, 20089 Milan, Italy; 10 Department of Medical and Surgical Sciences and Advanced Technologies “G.F. Ingrassia” Anatomic Pathology, University of Catania, 95123 Catania, Italy

**Keywords:** intravascular NK/T-cell lymphoma, Epstein–Barr virus, extranodal NK/T-cell lymphoma, aggressive NK-cell leukemia, EBV-positive primary nodal T/NK-cell lymphoma

## Abstract

**Simple Summary:**

Intravascular lymphoma is a neoplasm with tumor cells localized exclusively within blood vessel lumina. Most cases are of B-cell origin; only rare cases of NK or T-cell lineage. Unlike intravascular large B-cell lymphoma, which is a well-recognized entity in the WHO classification, intravascular NK/T-cell lymphoma is not considered a specific entity and there is still a debate about whether it is closer to aggressive NK leukemia or to extranodal NK/T-cell lymphoma. Our aim was to summarize the clinical, pathological, and molecular data on intravascular NK/T-cell lymphoma, which is a challenge both in terms of diagnosis and treatment. Recent molecular studies improved our understanding of the mechanism of lymphomagenesis, showing that multiple genetic events associated with EBV infection are required for the pathogenesis of this aggressive lymphoma. Promising therapeutic results may be offered by immune checkpoint inhibitors due to the high PD-L1 expression, which is possibly related to EBV infection.

**Abstract:**

Intravascular lymphoma is a form of lymphoid malignancy characterized by neoplastic cells growing almost exclusively within the lumina of small- to medium-sized blood vessels. Most cases are of B-cell origin with rare cases of natural killer or T-cell lineage. Extranodal sites are affected, mainly the skin and central nervous system, although any organ may be involved. Intravascular NK/T-cell lymphoma deserves special attention because of its clinicopathologic features and the need for adequate immunophenotyping combined with clonality test for a proper diagnosis. Moreover, intravascular NK/T-cell lymphoma is strongly linked to Epstein–Barr virus (EBV), which is considered to play a role in tumorigenesis and to be responsible for the aggressive behavior of the disease. In this paper, we review the current knowledge on this rare lymphoma and, in particular, the most recent advances about its molecular landscape. The main distinguishing features with other EBV-related entities, such as extranodal NK/T-cell lymphoma, EBV-positive primary nodal T/NK-cell lymphoma, and aggressive NK-cell leukemia, are discussed to help pathologists obtain the correct diagnosis and consequently develop an adequate and prompt therapy response.

## 1. Introduction

Intravascular lymphoma (IVL) is a malignancy characterized by the presence of tumor cells localized within blood vessel lumina. Although IVL may involve any organ, the majority of cases are reported within the skin and central nervous system (CNS).

IVL is more often of B-cell nature. IVL of T and NK-cell lineage is extremely rare, with a limited number of cases with sufficient immunohistochemical and clonality data reported so far [1,2,3,4,5,6,7,8,9,10,11,12,13,14,15,16,17,18,19,20,21].

In the last World Health Organization (WHO) classification of lymphoid neoplasms, intravascular NK/T-cell lymphoma (IVNKTL) is not recognized as a specific entity, but as very close to extranodal NK/T-cell lymphoma (ENKTL), from which it differs for its typical intravascular nature [22].

ENKTL is a high-grade lymphoma involving at presentation, in the majority of cases, the nasal or upper aerodigestive tract and, more rarely, other non-nasal, extranodal sites, including the skin [23,24,25,26].

The disease is prevalent in adult males from Asia and Latin America. EBV infection and genetic predisposition are involved in the pathogenesis of ENKTL, therefore explaining the geographic distribution of the neoplasm [22,26,27,28].

Whereas ENKTL is a mass-forming malignancy, classically characterized by the presence of neoplastic cells infiltrating the tissues with an angioinvasive and angiodestructive pattern of growth, IVNKTL does not form any mass and is characteristically a blood-vessel lumina-restricted lymphoma, affecting predominantly skin and CNS [18].

In the recently published review article by Alaggio et al., IVNKTL, due to its unclear nosological nature, is now described under aggressive NK-cell leukemia (ANKL) rather than ENKTL, although further studies are required to define where it fits best [29].

The present paper highlights the clinicopathologic features of IVNKTL, focusing on the main differential diagnoses as well as on the molecular pathogenesis of this rare, highly aggressive disease. Due to its relation to EBV infection, a brief introductive paragraph on EBV biology is also included.

## 2. Overview on EBV Biology

EBV, also known as human herpes virus 4 (HHV4), is one of the most diffuse human viruses [30,31,32,33]. The majority of adults are estimated to be seropositive.

People generally get infected at an early age through saliva. The virus entries through the oropharynx and, during a lytic phase, it replicates in the oropharyngeal epithelial cells. During this initial lytic phase, the host lacks immunity against EBV, whereas in the following weeks, cellular and humoral immunity develop, and the viral genome remains in a latent phase within memory B-lymphocytes.

Primary infection may be either asymptomatic or lead to infectious mononucleosis (IM), often with a limited course. The EBV enters B lymphocytes through the binding between the major viral envelope glycoprotein gp350 and the complement receptor 2 (CR2/CD21) on the B-lymphocyte surface. Moreover, the viral glycoprotein (gp42) acts as a cofactor binding to human leucocyte antigen (HLA) class II molecules on B-lymphocytes.

Memory B-cells are the virus reservoir in healthy people; hence, through this hiding mechanism (latency), the virus may elude the immune system. EBV-infected cells express, in different latency phases, the following six types of EBV nuclear antigen (EBNA): EBNA1, EBNA2, EBNA3A, EBNA3B, EBNA3C, and EBNA leader protein and three types of latent membrane protein (LMP): LMP1, LMP2A, and LMP2B. The latency phase of EBV infection is sub-classified into types III, II, and I, the former progressing to the latter. Upon infection of resting naïve lymphoid B-cells, EBV enters into type-III latency, in which all viral gene products are expressed, leading to the production of immunogenic viral proteins and causing a strong reaction of the T-cell immunity. Subsequently, the virus enters type-II latency with restriction of gene viral expression; latency II is characterized by expression of to EBNA1, LMP1, and LMP2, and in this phase, B-lymphocytes differentiate into memory B-cells. Finally, in type-I latency, viral gene expression is further restricted, with all infected cells expressing only EBNA1, which is responsible for the maintenance and replication of the EBV viral genome. Since approximately 50 years ago, when EBV was discovered, the virus has been shown to play an oncogenic role in different neoplasms, both lymphomas, and epithelial tumors—for instance, gastric carcinoma and nasopharyngeal carcinoma.

Interestingly, EBV may infect not only B-lymphocytes but even T-lymphocytes and NK-cells, although the mechanism for virus entrance into T/NK-cells is not well-known [34,35,36,37,38].

EBV-associated diseases develop predominantly in immunocompromised individuals when the balance between the immune system of the host and the infection is altered. EBV plays a pathogenetic role in lymphoproliferative disorders (LPDs) of B-cell origin, as well as of T- and NK-cell origin, including IVNKTL [28,39,40].

## 3. Overview on the PD-1/PD-L1 Axis and Its Relation to EBV in Lymphomas

The PD-1 pathway, which includes the immune cell co-receptor programmed death 1 (PD-1) and its ligands, programmed cell death ligand 1 (PD-L1) and programmed cell death ligand 2 (PD-L2), acts on local immunosuppression in the tumor microenvironment (TME) [41,42,43].

PD-1 is expressed on immune cells such as T-, B-, and NK-cells; PD-L1 is expressed on neoplastic cells and antigen-presenting cells, and PD-L2 is displayed by activated monocytes and dendritic cells.

The survival of several neoplasms is favored by the PD-1/PD-L1 interaction; PD-L1 expressed by tumor cells binds with its receptor PD-1, displayed by immune cells, and favors the process of immune evasion by neoplastic cells.

The PD-1/PD-L1 interaction suppresses the activity and proliferation of cytotoxic tumor-infiltrating lymphocytes (TILs) and favors the immunosuppressive effects of regulatory T-lymphocytes. Additionally, the PD-1/PD-L1 binding has multiple effects on all the components of the TME that favor tumor immune evasion, for instance, regulating the secretion of cytokines and the inhibition of NK-cells, which may directly eliminate neoplastic cells [41,42,43].

PD-1/PD-L1 inhibitors represent a highly promising cancer treatment option because, by blocking PD-1–PD-L1 signaling, they can prevent tumor immune escape and facilitate a positive immune response to kill tumor cells.

Currently, checkpoint inhibitors represent a frontline therapy for several neoplasms such as non-small-cell lung cancer, metastatic melanoma, renal cell carcinoma, urothelial tumors, and head-and-neck squamous cell carcinoma, but they are also under active evaluation in many other solid cancers, as well as in some hematological neoplasms [44,45,46].

EBV is associated with a variety of lymphomas, and immunodeficiency has always been considered to play a critical role in the development of EBV-related lymphomas. However, the immune escape of tumor cells has recently been regarded as contributing to the development of some EBV-associated hematological malignancies, in which PD-L1 overexpression is observed [45].

Upregulation of PD-L1 is found in EBV-associated NK/T-cell lymphomas, which are the subject of the present review, and EBV infection is considered to play an important role in upregulating PD-1/PD-L1 expression through various mechanisms (better detailed in the paragraphs of the different entities). In ENKTL, both LMP1 overexpression and the *JAK/STAT* pathway contribute to PD-L1 overexpression and to tumor immune evasion.

Recently, promising results have been obtained with the use of checkpoint inhibitors in lymphomas; in particular, lymphomas characterized by immune escape of the tumor cells are considered good candidates for anti-PD-1/PD-L1 therapy [45,46,47].

## 4. Clinicopathological Features of IVNKTL

IVNKTL is an exceedingly rare lymphoid malignancy with a limited number of cases (approximately 22) with sufficient immunophenotypic and molecular data reported in the literature [1,15,16,17,19,20,21]. The disease occurs predominantly in adults with a variable range of ages; the skin and CNS are the most frequently involved sites, with the skin being the most frequent and, in some cases, the unique site affected. Tender erythematous subcutaneous nodules are the most frequent clinical manifestation [14].

Notably, even in IVL of B-cell origin, which is far more frequent than IVNKTL, the skin is the main organ affected, being involved alone or in combination with other organs—the CNS predominating, in 40% of cases [7,22]. Intravascular large B-cell lymphoma (IVLBL), which is a well-recognized entity, has two distinct patterns of presentation: the Western variant, mainly affecting skin and CNS, and the Asian variant, usually involving multiple organs and often associated to hemophagocytic lymphohistiocytosis (HLH) and worse prognosis [48].

Nonetheless, considering the almost-exclusive blood-vessel lumina-restricted growth pattern of IVNKTL without mass lesions, prior to post-mortem evaluation, it can be presumed that almost any organ may be affected [18].

The clinical manifestations are multiple, including neurologic symptoms, fever, weight loss, malaise, arthralgia, jaundice, and night sweats. As the clinical picture is rather confusing and tumor masses are not detectable by imaging examination, making the correct diagnosis is particularly challenging. Bone marrow (BM) involvement may cause hematologic symptoms such as anemia, leucopenia, and thrombocytopenia; however, BM intravascular involvement can be subtle and difficult to detect in absence of adequate immunohistochemical and molecular evaluations [2,13,18].

**Figure 1:**
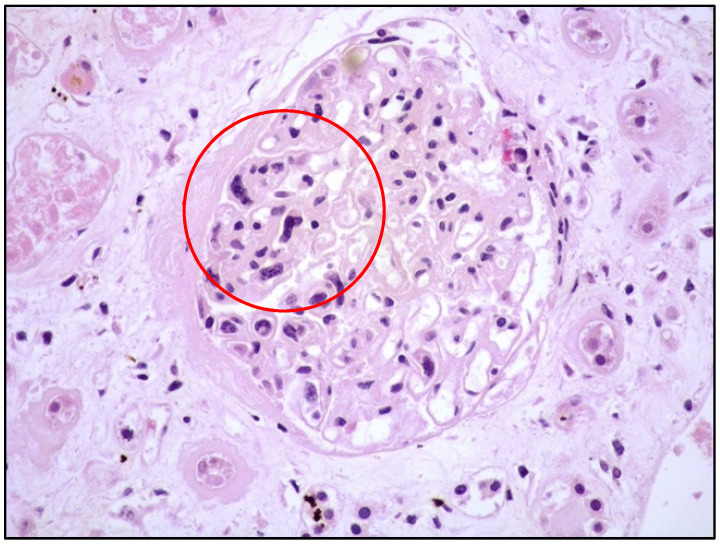
High-power view showing kidney parenchyma with large, atypical cells growing within vascular spaces (see within red circle; hematoxylin and eosin, 200× magnification, previously unpublished, original image from S.A.).

**Figure 2:**
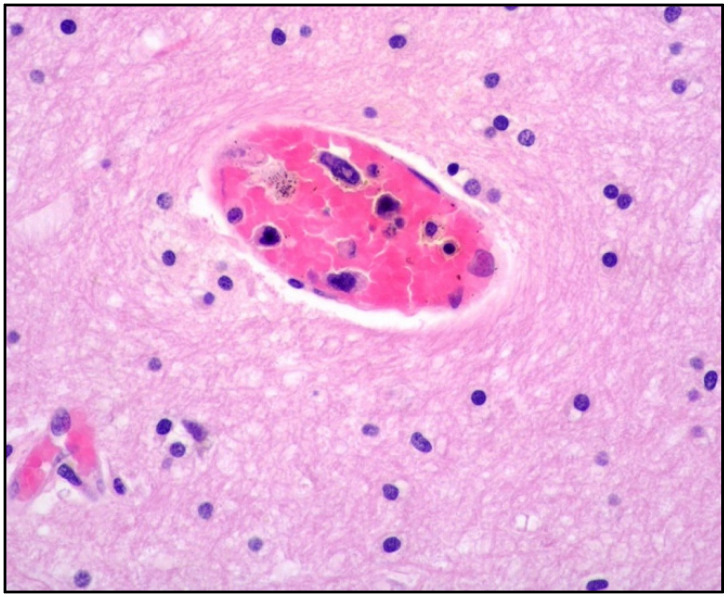
High-power view showing central nervous system parenchyma with atypical cells within a vascular space (hematoxylin and eosin, 400× magnification, previously unpublished, original image from S.A.).

Histologically, the tumor cells are usually discohesive, pleomorphic, and large-sized, with scant cytoplasm and irregular, enlarged nuclei showing hyperchromatic nucleoli; the cells grow inside the lumina of small- to medium-sized vessels without forming a mass (Figure 1 and Figure 2).

**Figure 3:**
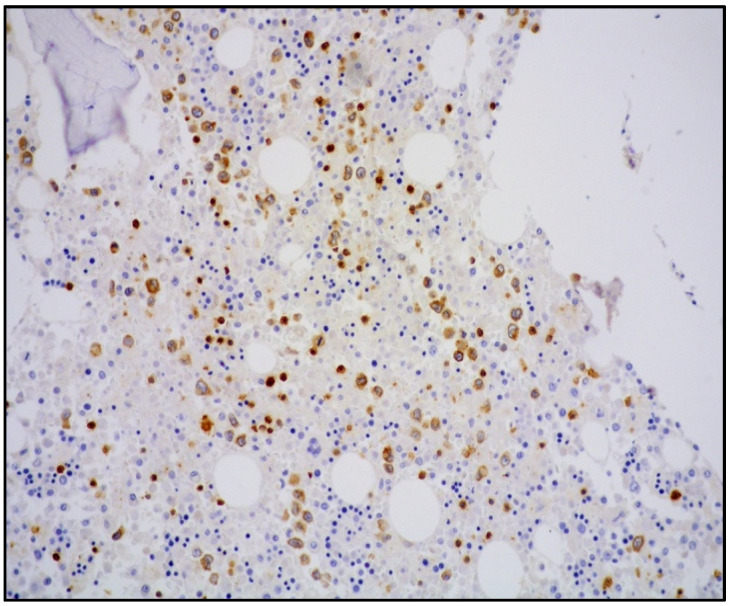
Medium-power view showing bone marrow with CD3-positive atypical cells within sinusoidal spaces (CD3 immunostaining, 100× magnification, previously unpublished, original image from S.A.).

**Figure 4:**
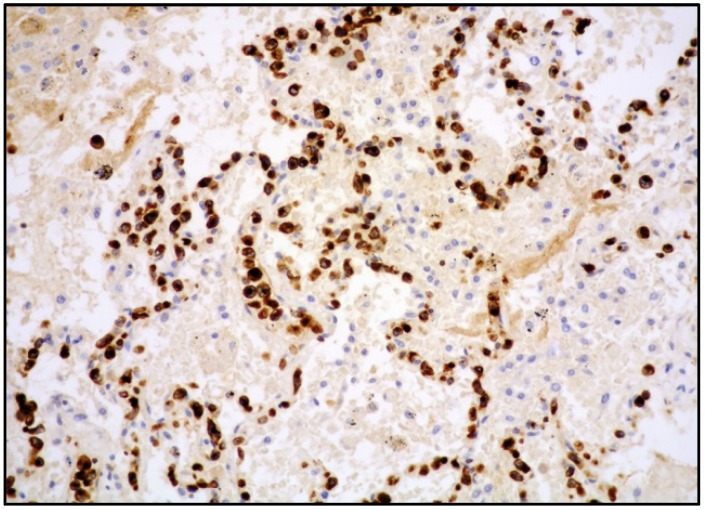
High-power view showing lung parenchyma with perforin-positive atypical cells within vascular spaces (perforin immunostaining, 200× magnification, previously unpublished, original image from S.A.).

The majority of NK/T-cell IVL express either T-cell (Figure 3) or NK-cell markers (CD56) and cytotoxic molecules such as granzyme B and perforin (Figure 4).

**Figure 5:**
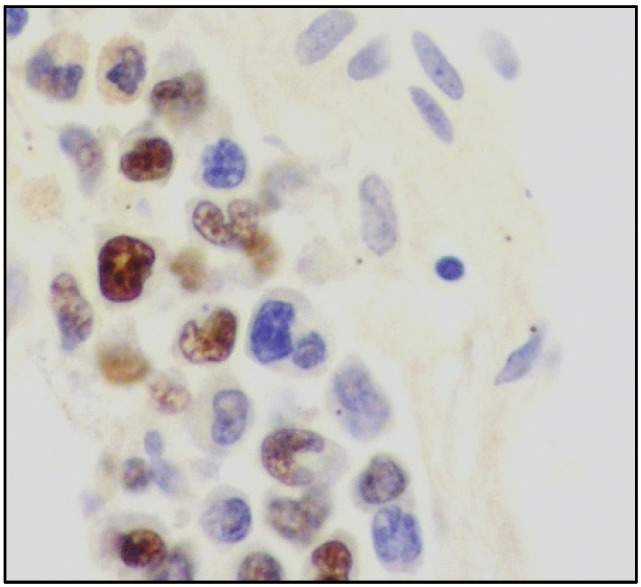
High-power view showing lung parenchyma with EBER-positive atypical cells within vascular spaces (in situ hybridization for EBV-encoded RNA, 400× magnification, previously unpublished, original image from S.A.).

In the majority of cases, there is a strong association with EBV infection and diffuse positive staining with in situ hybridization for EBV-encoded RNA (EBER) (Figure 5).

In IVNKTL, the precise lineage assignment requires accurate phenotypic analyses to differentiate cases with T-cells from those with NK-cell origin; however, differentiation may be difficult, as NK-cells can be positive for markers also expressed by T-lymphocytes, such as cytoplasmic CD3 (cCD3), CD2, CD7, CD8, CD56, and cytotoxic antigens. NK-cells are generally negative for CD5; T-cell clonality is the best way to define the cell of origin as the T-cell receptor (TCR) genes are not rearranged in true NK-cell proliferations [22].

Incorporating analysis from the literature of the immunophenotypic data for this lymphoma is complicated because of the different antibodies used, which are often insufficient for a precise judgement, particularly in older reports.

Although the outcome of patients with clinical manifestations limited to the skin appears better than that of patients with multiple organ involvement, according to the literature data, this difference is not statistically significant, and IVNKTL remains an aggressive disease with poor response to chemotherapy [14].

Yan et al. reported an improvement in survival of 11 months in patients who received chemotherapy compared with non-chemotherapy patients [14]. No standard chemotherapy regimen is currently defined for the disease. A traditional CHOP-based regimen (cyclophosphamide, doxorubicin, vincristine, prednisolone) seems insufficient for IVNKTL. CHOP plus stem cell transplantation, salvage chemotherapy with DHAP (dexamethasone, cytarabine, cisplastin), and proteasome inhibitor treatment have been found to be more effective in some patients [14]. As discussed in the following paragraph, the disease may be susceptible to immune checkpoint inhibitor drugs due to the strong expression of PD-L1 in IVNKTL [19].

## 5. Molecular Features and Pathogenesis of IVNKTL

In 2019, Fujikimura et al. analyzed the somatic mutations and copy number alterations (CNAs) of two cases of IVNKTL using whole-exome sequencing (WES) analysis [19] and found frequent alterations in genes encoding epigenetic regulators. Interestingly, four histone genes (*HIST1H2AN*, *HIST1H2BE*, *HIST1H2BN*, and *H3F3A*) were mutated in both cases, making histone genes as frequently mutated in this type of lymphoma.

In addition, mutations in *TET2* or *DNMT1*, which generally defend against abnormal DNA methylation in the genome, were found, and it is well-known that DNA methylation alteration is a major oncogenic event in some lymphomas [49].

Fujikimura et al. also found a strong expression of PD-L1 in IVNKTL [19]. Recent studies support the role of EBV infection in upregulating PD-1/PD-L1 expression in various neoplasms [50]. Therefore, the high levels of PD-L1 expression identified in IVLNKL may be strictly related to EBV infection, as occurs in other neoplasms with EBV-infected lymphoid cells. The EBV oncoprotein LMP1 possibly drives PD-L1 overexpression in IVNKTL as in ENKTL [19].

It is now well-known that neoplasms that are strongly positive for PD-L1 show a high rate of response to PD-1/PD-L1 blockade, which may therefore represent a potentially effective treatment in this type of lymphoma [51,52,53,54,55].

This first study by Fujikimura et al. was mainly on somatic alterations occurring at the DNA level and supported the concept that multiple genetic events associated with EBV infection are required for the pathogenesis of this lymphoma [19].

More recently, in 2020, Fujikimura’s group addressed the complex issue of the IVNKTL transcriptome [21].

It is also well-known that the human genome contains about 20,000 protein-coding genes, whereas their transcriptome is much more complex, containing 83,666 distinct mRNA sequences—at least in the currently used version of GENCODE [56]. The relevant discrepancy between the number of protein-coding genes and the number of mRNA sequences is understandable, considering the role of the process of alternative splicing (AS).

AS is responsible for splicing single mRNA precursors into different arrangements, producing proteins with distinct structures and functions; therefore, AS has a remarkably important effect on the biological features of the final protein [57].

AS is frequently altered in malignancies, playing an important role in tumorigenesis as well as in tumor progression [58,59,60,61,62,63,64].

Using high-throughput RNA sequencing analysis, the authors evaluated two cases of IVNKTL for the tumor-related modifications that occur at the stages of transcription and splicing and identified transcript isoforms specific to this lymphoma [21].

In this study, the authors identified AS behavior that is known to be tumor-specific in cancer driver genes involved in IVNKTL origin, such as *HRAS*, *MDM2*, *FGFR2*, and *VEGFA* [21].

The whole transcriptome analysis supported the hypothesis that this lymphoma may be driven by multiple factors, including non-exonic transcription, defects in epigenetic regulators, AS, and EBV infection [21].

## 6. Differential Diagnoses of IVNKTL

### 6.1. ENKTL, ANKL, and EBV-Positive Nodal T/NK Lymphoma

ENKTL and ANKL are EBV-positive T- or NK-cell malignancies, with distinct clinical features. EBV-positive nodal T- (or, rarely, NK-) cell lymphoma is a recently recognized entity characterized by an EBV-positive tumor primarily involving the lymph nodes. These three entities have different clinical presentations but share EBV as an important etiologic factor and a cytotoxic phenotype of either T- or NK-cells. The main clinicopathologic features for the differential diagnoses are summarized in Table 1.

### 6.2. Extranodal NK/T-Cell Lymphoma (ENKTL)

#### 6.2.1. General Features and Clinical Manifestations

Extranodal NK/T-cell lymphoma (ENKTL) is an EBV-associated NK- or cytotoxic T-cell lymphoma occurring at extranodal sites [22,23,24,25,26,27,28].

The majority of cases (approximately 80%) are represented by what is called the nasal form, in which the sites of presentation are the nasal or upper aerodigestive tract; however, even in the nasal form, systemic involvement of other extranodal sites may occur at advanced stages of disease. Primary nodal involvement is extremely rare, whereas lymph nodes may be involved secondarily.

A minority of cases (approximately 20%) are represented by the non-nasal form, arising in extranodal, non-nasal sites [22,65,66].

ENKTL has a particular ethnic distribution, mainly affecting adult males from Asia and Latin America. EBV infection is strongly linked to ENKTL with virtually all cases positive for EBV. Hence, EBV-positivity is considered a diagnostic criterion, although it is not completely clear how the virus plays its oncogenic role; the overexpression of LMP1 protein, as well as the activation of the NF-kB signaling pathway, may be possible EBV-associated oncogenic mechanisms [26,27].

The most common clinical manifestations of the nasal form are nasal obstruction, epistaxis, and often destructive lesions involving the upper aerodigestive tract and midface.

As already mentioned, non-nasal sites such as skin, soft tissue, testis, salivary gland, liver, spleen, and gastrointestinal tract may be the primary sites of involvement, causing clinical manifestations varying according to the sites affected.

BM involvement may occur during the course of the disease, and HLH syndrome represents a serious complication often developing in cases with BM involvement [67]. Systemic symptoms such as fever and weight loss may be present.

Grossly, ENKTL usually causes an ulcerated mass.

#### 6.2.2. Histology

The classic histological picture is characterized by a rich inflammatory background associated with necrosis sometimes overwhelming the neoplastic component and making difficult to distinguish ENKTL from an inflammatory condition.

The malignant lymphoid cells present as a diffuse proliferation with an angiocentric and angiodestructive pattern of growth and fibrinoid necrosis in the vessel wall.

Of note, when ENKTL involves skin or mucosal sites, the epithelium may show features of pseudoepitheliomatous hyperplasia, simulating squamous cell carcinoma [68].

**Figure 6:**
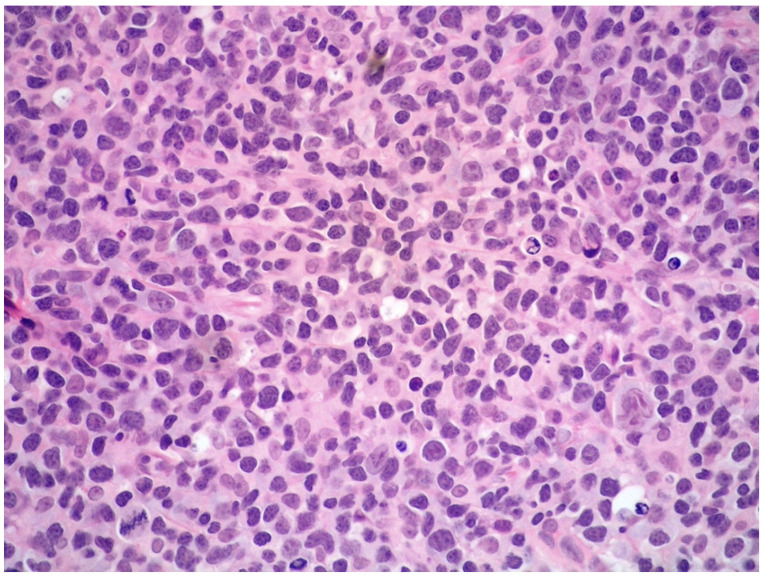
High-power view showing a diffuse and polymorphic proliferation of atypical, medium- to large-sized lymphoid cells (hematoxylin and eosin, 200× magnification, previously unpublished, original image from S.A.).

The neoplastic elements are rather polymorphic and variable in size, although small- to medium-sized cells with scarce and clear cytoplasm, irregular nucleus, granular chromatin, and inconspicuous nucleolus are generally prevalent. Large-sized lymphoid cells may be present. Mitotic figures are frequent. (Figure 6).

#### 6.2.3. Immunophenotypic Features

**Figure 7:**
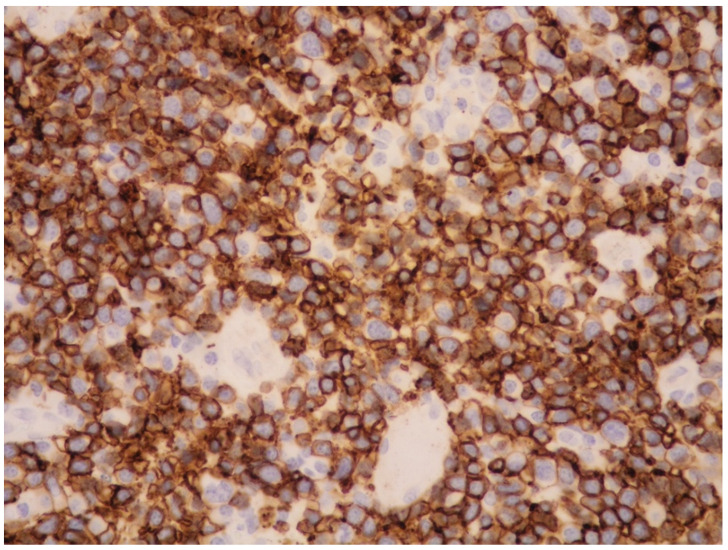
High-power view showing a diffuse proliferation of CD56-positive atypical cells (CD56 immunostaining, 200× magnification, previously unpublished, original image from S.A.).

The disease may be of NK- or T-cell nature [69], although it is more often of NK-cell origin with neoplastic elements positive for cytotoxic molecules (TIA1, perforin, and granzyme B), CD2, cCD3, CD56 (Figure 7).

**Figure 8:**
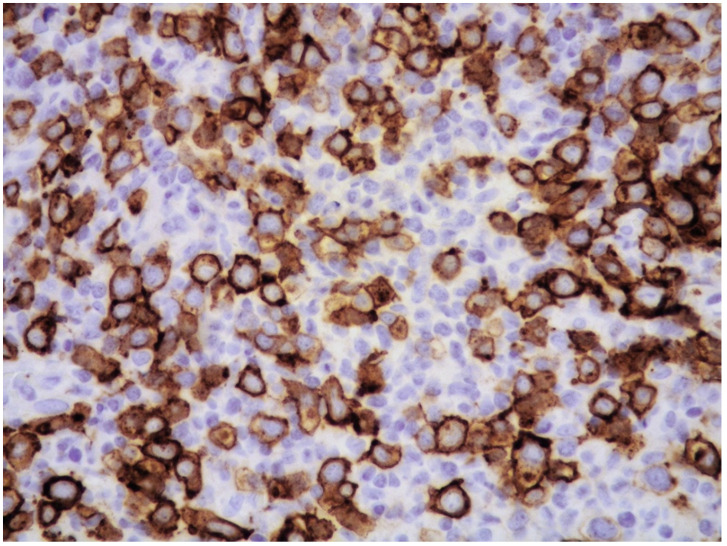
High-power view showing CD30-positive atypical cells (CD30 immunostaining, 400× magnification, previously unpublished, original image from S.A.).

CD7 and CD30 are rarely expressed, although, particularly in the non-nasal form, approximately 30% of neoplastic cells are positive for CD30 (Figure 8).

Surface CD3 (sCD3), CD4, CD8, CD5, CD16, and CD57 are usually negative, and the T-cell receptor gene (TCR) rearrangement is negative in the form of NK-cell origin.

A minority of cases (approximately 40%) belonging to the cytotoxic T-cell lineage are positive for sCD3, CD5, and CD8 and show T-cell clonality. Nuclear expression of megakaryocyte-associated tyrosine kinase (MATK) is frequent [69].

**Figure 9:**
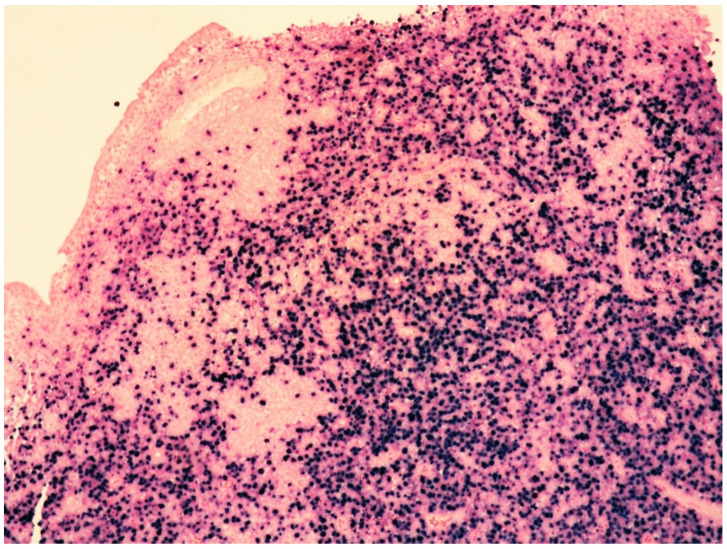
Medium-power view showing a diffuse proliferation of EBER-positive cells (in situ hybridization for EBV-encoded RNA, 100× magnification, previously unpublished, original image from S.A.).

EBER is positive in the majority of viable neoplastic cells (Figure 9).

Type-II latency pattern with neoplastic cells often expressing LMP1, LMP2, and EBNA2 is often found.

CD56 is a useful, but not specific, marker for NK-cells, and most ENKTL are CD56-positive; however, CD56 can be negative in approximately 20% of cases, especially in ENKTL of T-cell origin [69]. To diagnose a CD56-negative lymphoma as ENKTL, the neoplasm must express a cytotoxic molecule and EBV.

As already mentioned, other NK-cell markers, such as CD16 or CD57, are commonly negative. As ENKTL of T and NK-cell origin may share the expression of some cell markers, the cell lineage cannot be distinguished only by immunophenotyping, but it is determining the identification or not of T-cell clonality. Rarely, the aberrant expression of B-cell markers such as CD20 may be found [70].

In ENKTL, PD-1 is rarely expressed, whereas PD-L1 is frequently positive [71]. PD-L1 is the ligand of the immune-checkpoint protein PD1 identified on T-lymphocytes. By binding to PD1 on T-lymphocytes and blocking their functions, PD-L1 favors the mechanism of immune evasion by neoplastic cells of ENKTL [72].

As previously mentioned, an association between EBV and the PD1/PD-L1 pathway has been noted in different neoplasms, and the overexpression of PD-L1 represents a mechanism of immune evasion in lymphoid cells infected by EBV [72].

In ENKTL, PD-L1 overexpression is possibly related to different factors. The EBV-related oncoprotein LMP1 is one of the mechanisms driving PD-L1 overexpression through the *NF-kB* pathway [72].

In ENKTL, both *JAK/STAT* and *NF-kB* are usually activated, and multiple recurrent genetic and epigenetic (hypermethylation) alterations have been identified [24,27,73,74,75,76]. The *JAK/STAT* pathway also contributes to PD-L1 overexpression by interacting with the interferon-stimulated response element in the promoter region of PD-L1 [72].

#### 6.2.4. Genetic Features

ENKTL shows multiple genomic alterations. Deletion of 6q21-25 is the most identified abnormality, harboring several tumor suppressor genes (*PRDM1*, *PTPRK*, *FOXO3*, *HACE1*). *PRDM1* is a tumor suppressor gene, often found to be inactivated by mutation, deletion, or hypermethylation, which plays an important role in ENKTL pathogenesis [77].

Mutations of tumor suppressor genes such as *BCOR*, *DDX3X*, and *TP53* have also been identified [77].

#### 6.2.5. Treatment

ENKTL is notoriously resistant to anthracycline-based chemotherapy, whereas the use of L-asparaginase-based chemotherapy combined with radiotherapy has improved the outcome for this aggressive lymphoma [78]. However, as chemotherapy is often unable to achieve long-term remissions in advanced stages of the disease, it is extremely important that novel therapies targeting different molecular pathways are currently available [79,80].

Despite still lacking a standardized expression cutoff for predicting the response to PD-1/PD-L1 inhibitors, several clinical trials are ongoing, and immune checkpoint inhibitor therapy is used in relapsed or refractory disease [81]. Due to the relevant role played by the activation of both *JAK/STAT* and *NF-kB* pathways in ENKTL pathogenesis, these pathways are potentially targetable for therapy [74,75,76,79,80]. Further studies are needed to evaluate the efficacy of the anti-CD30 antibody brentuximab vedotin, particularly in combination with chemotherapy, for the treatment of ENKTL [82].

In brief, the main clinicopathologic differences between IVNKTL and ENKTL are the sites of involvement, as the nasal and upper aerodigestive tract are the sites most involved in ENKTL, whereas IVNKTL frequently involves the skin and CNS with no nasal involvement; a further difference is the presence of mass lesions in ENKTL, whereas IVNKTL usually develops within vascular spaces in the absence of a clear-cut mass.

### 6.3. EBV-Positive Nodal T/NK-Cell Lymphoma

#### 6.3.1. General Features and Clinical Manifestations

EBV-positive nodal T/NK-cell lymphoma is a primary nodal EBV-positive lymphoma that usually exhibits a cytotoxic T-cell phenotype or, more rarely, one of NK-cell nature. By definition, it is a systemic disease primarily affecting lymph nodes with the involvement of a limited number of extranodal sites and, in particular, lacking nasal involvement.

In the 2017 WHO classification, it was listed as an EBV-positive variant of peripheral T-cell lymphoma, not otherwise specified (PTCL, NOS), whereas in the 2022 International Consensus Classification of Mature Lymphoid Neoplasms (2022 ICC), as well as in the recent article by Alaggio et al., it is recognized as a new provisional entity distinct from PTCL, NOS [22,29,83,84,85].

Patients are mainly adults/elderly from East Asia, and the neoplasm may occur in the setting of immunosuppression or in association with autoimmune diseases. Patients present with multiple lymphadenopathy with or without extranodal involvement such as hepatosplenomegaly, B symptoms, and advanced stages. The outcome is poor.

#### 6.3.2. Histology and Immunophenotypic Features

The morphological features are those of a high-grade lymphoma, either monomorphic or polymorphic. In the majority of cases (approximately more than 80%), this neoplasm is of T-cell lineage, based on T-cell clonality, with less than 20% of cases being of NK-cell origin [86,87].

The neoplastic cells are usually positive for T-cell markers and cytotoxic molecules. The typical phenotype is CD8-positive, CD56-negative, cytotoxic αβT cells. A minority of cases (0–13%) show γδT cells, whereas αβT cells are expressed in 40–60% of cases [87].

EBV positivity is a diagnostic criterion, and the virus plays an important pathogenetic role. Similarly, in ENKTL and other EBV-associated T/NK lymphoproliferative diseases, most cases of EBV-positive nodal T/NK-cell lymphoma have a type-II EBV latency pattern and EBNA1, LMP1, and LMP2A are expressed, although LMP1 protein is difficult to find [88,89].

Histologically, unlike ENKTL, this lymphoma usually shows a monomorphic pattern of growth, the absence of necrosis and angioinvasion, and infrequent CD56 expression.

#### 6.3.3. Genetic Features

The genetic profile is different from that of ENKTL, with the most mutated gene being *TET2* (64%) [89]. Other common mutated genes are *PIK3CD* (33%), *DDX3* (20%), and *STAT3* (19%).

Recently, this lymphoma has been found to be characterized by low genomic instability, upregulation of immune-related pathways favoring tumor evasion, and downregulation of EBV miRNA [89]. This rare type of lymphoma, despite its aggressive behavior, has been demonstrated to be more genomically stable than ENKTL and PTCL, NOS, supporting its consideration as a distinct entity [89]. The downregulation of EBV miRNA observed in EBV-positive nodal T/NK-cell lymphoma compared to ENKTL suggests that there are differences in the EBV biology between these diseases.

The main clinicopathological differences between EBV-positive nodal T/NK-cell lymphoma and IVNKTL are the sites of presentation, as IVNKTL mainly has an extra-nodal presentation and rarely occurs primarily in lymph nodes. In addition, in EBV-positive nodal T/NK-cell lymphoma, intravascular involvement is rare, cells are more often of T-cell origin, and CD56 is often negative.

### 6.4. Aggressive NK-Cell Leukemia (ANKL)

#### 6.4.1. General Features and Clinical Manifestations

ANKL is a rare and aggressive leukemia, more prevalent in Asia, NK-cell in origin and characterized by a strong association with EBV, identified in about 90% of cases. EBV is considered to play an important role in the pathogenesis of ANKL and it is considered to be responsible of the aggressive nature of the disease.

Unlike IVNKTL, ENKTL, and EBV positive nodal T/NK-cell lymphoma, ANKL is a malignancy of young to middle-aged adults [90,91]. Particularly in young individuals, the disease may represent an aggressive evolution of chronic active EBV disease (CAEBV) [92]. Rare cases of EBV-negative ANKL have been described either developing de novo or deriving from NK-large granular lymphocyte leukemia (LGLL) [93,94].

Commonly involved sites are the BM, peripheral blood (PB), liver, and spleen, but any organ may be involved [90]. The number of leukemic cells in PB and BM smear is variable and may be low.

Patients present with fever, a leukemic blood picture, and symptoms of HLH syndrome. Hepatosplenomegaly and disseminated intravascular coagulation are frequently observed. Lymphadenopathy may be present, whereas skin involvement is rare. The lack of nasal involvement is a distinguishing feature from ENKTL. The disease usually follows a fulminant clinical course, although it is not clear whether the very rare EBV-negative cases have the same poor prognosis [94].

#### 6.4.2. Histology and Immunophenotypic Features

Histologically, neoplastic elements vary from normal large granular lymphocytes (LGLs) to highly atypical cells, with large, irregular nuclei and evident nucleoli. The cells may have pale cytoplasm or contain azurophilic granules. Malignant cells can involve the bone marrow with an interstitial or sinusoidal pattern of growth, and the involvement can be either subtle or massive.

The immunophenotype is identical to that of ENKTL, except for CD16 expression, which is far more frequent (75%) in ANKL compared to ENKTL (22%).

#### 6.4.3. Genetic Features

Some genetic abnormalities such as el(6)(q21q25), i(7)(q10) and 11q deletion have been found in ANKL [95]. Nakashima et al. found different genomic alteration patterns in ANKL and ENKTL, with loss in 6q being more frequent in ENKTL, unlike losses in 7p and 17p and gains in 1q more frequently present in ANKL [96]. ANKL has been found to harbor mutations in *TP53* (34%), *TET2* (28%), *CREBBP* (21%), and *MLL2* genes (21%) [96]. Mutations in the *JAK/STAT* pathway have been detected in 48% of cases [97], and Huang et al. found that ANKL cells show increased expression of the transcription factor *MYC,* which is under the control of *JAK* and *STAT* genes [98].

#### 6.4.4. Treatment

ANKL is a disease with a poor prognosis and is resistant to anthracycline-based chemotherapy. Treatments may consist of concurrent chemoradiation, sequential chemoradiation, or sandwich chemoradiation. The efficacy of L-asparaginase has led to its inclusion in different combination regimens. Treatments providing lower dose intensity of chemoradiation followed by ASCT have demonstrated some benefits. Relapse remains an issue in this disease, and novel treatments targeting molecular pathways such as the *JAK/STAT* pathway are currently under evaluation [97].

Regarding the relation between ANKL and IVNKTL, Alaggio et al. recently proposed that IVNKTL is more similar to ANKL than ENKTL in its intravascular presentation, however, the authors suggested that further studies are needed to better determine IVNKTL categorization [29].

## 7. Conclusions

IVNKTL is a rare lymphoma characterized by the exclusive growth of large cells within the lumen of small- and medium-sized vessels, involving mainly skin and CNS, although virtually any organ may be affected. It is strictly linked to EBV infection and needs an accurate differential diagnosis with other EBV-related NK/T-lymphomas. IVNKTL shows somatic mutations in epigenetic regulator genes. The high PD-L1 expression, probably due to EBV infection, suggests that checkpoint inhibitors may be effective treatments for this aggressive lymphoma.

Although WES analysis and transcriptome studies have provided new insight into molecular pathogenesis and possible treatment, the nature of IVNKTL is not completely elucidated. It has been recently postulated that IVNKTL may be more similar to ANKL than ENKTL.

Further studies on a larger number of cases with detailed immunophenotypic and molecular data are needed to clarify this issue.

## Figures and Tables

**Table 1:** Differential diagnoses of IVNKTL.

	IVNKTL	ENKTL	ANKL	EBV + Nodal T/NK Lymphoma
**Median Age and Sex**	Adult (middle-age, around 46 yrs) M > F	Adults (elderly, around 60 yrs) M > F	Adult (mainly young adult) M = F	Adult (middle-age, often immunocompromised) M = F
**Ethnicity**	Asian and Latin America	Asian	Asian	Asian
**Sites**	Skin and CNS (mainly); any organ may be involved	Extranodal sites (mainly nasal region and upper aerodigestive tract), very rare primary nodal sites	BM, blood, liver, spleen; very rare nodal presentation	Commonly nodal presentation
**Nasal involvement**	No	Very common	No	No
**Systemic symptoms**	Protean clinical manifestations	B-symptoms	B-symptoms; often CID	B-symptoms
**Tumor mass**	No	Yes, necrotic/ulcerated mass	No	Yes
**Histology**	Large-sized cells within medium- to small-sized vessels	Polymorphic cells, clear cytoplasm; inflammatory background, necrosis. Angioinvasive and angiodestructive pattern.	Variable in size from normal granular lymphocytes to large, atypical cells	High-grade morphology (either polymorphic or monomorphic)
**Cell of origin**	NK or cytotoxic T cells	NK > cytotoxic T cell	NK	Cytotoxic T-cell > NK
**EBV-association**	Common	Common	Common	Common
**CD56 expression**	Often positive	Often positive	Often positive (p16 generally positive)	Often negative
**T-cell clonality**	Present in cases of T-cell origin	Present in cases of T-cell origin	No	Present in cases of T-cell origin
**Prognosis**	Poor	Poor	Poor	Poor

Legends: ANKL: aggressive NK leukemia; BM: bone marrow; CID: coagulation intravascular disseminated; CNS: central nervous system EBV: Epstein–Barr virus; ENKTL: extranodal NK/T-cell lymphoma; F: female; IVNKTL: intravascular NK/T-cell lymphoma; M: male; yrs: years.
